# Xylooligosaccharide supplementation alters gut bacteria in both healthy and prediabetic adults: a pilot study

**DOI:** 10.3389/fphys.2015.00216

**Published:** 2015-08-07

**Authors:** Jieping Yang, Paula H. Summanen, Susanne M. Henning, Mark Hsu, Heiman Lam, Jianjun Huang, Chi-Hong Tseng, Scot E. Dowd, Sydney M. Finegold, David Heber, Zhaoping Li

**Affiliations:** ^1^Center for Human Nutrition, David Geffen School of Medicine, University of California, Los AngelesLos Angeles, CA, USA; ^2^Infectious Diseases Section, VA West Los Angeles Medical CenterLos Angeles, CA, USA; ^3^Department of Statistics Core, David Geffen School of Medicine, University of California Los AngelesLos Angeles, CA, USA; ^4^MR DNA Molecular Research LPShallowwater, TX, USA; ^5^Department of Microbiology, Immunology and Molecular Genetics, University of California Los AngelesLos Angeles, CA, USA

**Keywords:** xylooligosaccharide, prediabetic, diabetes, gut, microbiota

## Abstract

**Background:** It has been suggested that gut microbiota is altered in Type 2 Diabetes Mellitus (T2DM) patients.

**Objective:** This study was to evaluate the effect of the prebiotic xylooligosaccharide (XOS) on the gut microbiota in both healthy and prediabetic (Pre-DM) subjects, as well as impaired glucose tolerance (IGT) in Pre-DM.

**Subjects/Methods:** Pre-DM (*n* = 13) or healthy (*n* = 16) subjects were randomized to receive 2 g/day XOS or placebo for 8-weeks. In Pre-DM subjects, body composition and oral glucose tolerance test (OGTT) was done at baseline and week 8. Stool from Pre-DM and healthy subjects at baseline and week 8 was analyzed for gut microbiota characterization using Illumina MiSeq sequencing.

**Results:** We identified 40 Pre-DM associated bacterial taxa. Among them, the abundance of the genera *Enterorhabdus, Howardella*, and *Slackia* was higher in Pre-DM. XOS significantly decreased or reversed the increase in abundance of *Howardella, Enterorhabdus*, and *Slackia* observed in healthy or Pre-DM subjects. Abundance of the species *Blautia hydrogenotrophica* was lower in pre-DM subjects, while XOS increased its abundance. In Pre-DM, XOS showed a tendency to reduce OGTT 2-h insulin levels (*P* = 0.13), but had no effect on body composition, HOMA-IR, serum glucose, triglyceride, satiety hormones, and TNFα.

**Conclusion:** This is the first clinical observation of modifications of the gut microbiota by XOS in both healthy and Pre-DM subjects in a pilot study. Prebiotic XOS may be beneficial in reversing changes in the gut microbiota during the development of diabetes.

**Clinical trial registration:** NCT01944904 (https://clinicaltrials.gov/ct2/show/NCT01944904).

## Introduction

Increasing evidence indicates that changes in gut microbiota composition might contribute to the development of metabolic disorders such as obesity and T2DM (Qin et al., 2012). Several mechanisms have been proposed regarding how gut bacteria could facilitate the pathogenesis of T2DM (Shen et al., 2013). Studies suggest that gut bacteria influence whole-body metabolism through regulation of the host's immune response, energy extraction and utilization, intestinal glucose absorption, and lipid metabolism (Musso et al., 2011). The central feature of obesity and T2DM is insulin resistance, potentially caused by low-grade inflammation resulting from nutrient excess and leading to endoplasmic reticulum (ER) stress. More and more research has shown that gut microbiota is another important factor for this low-grade inflammation (Chassaing and Gewirtz, 2014). The *Bacteroidetes*/*Firmicutes* ratio is associated with increased plasma glucose concentrations and a decrease in butyrate-producing bacteria in T2DM patients (Larsen et al., 2010; Shen et al., 2013). Recent studies in mice have demonstrated that an increase in the abundance of *Bifidobacteria* and *Akkermansia muciniphila* attenuated high fat diet-induced metabolic complications (Everard et al., 2013).

Pre-DM refers to the intermediate stage between normoglycemia and overt diabetes mellitus. Pre-DM is characterized by glucose dysregulation and a higher risk of developing T2DM and other associated complications (Portero McLellan et al., 2014). However, not all individuals with Pre-DM progress to overt T2DM. With changes in lifestyle and diet, the progression of Pre-DM to T2DM can be prevented or delayed (Portero McLellan et al., 2014). Given the significant involvement of certain gut bacteria in host metabolism (Shen et al., 2013), therapeutic manipulation of the gut microbiota has been proposed for both individuals with T2DM and in those at risk of developing the condition.

Prebiotics are highly effective and important for many applications in medicine. They are not digestible and do not contribute to human nourishment, but rather exert a profound effect on the human gut microbiota (International Scientific Association for Probiotics and Prebiotics, 2004). Prebiotic-induced modulation of gut microbiota has been developed and widely used (Everard et al., 2011). The principal effect of prebiotics on the human gut microbiota is to stimulate the growth of the *Bifidobacterium* and *Lactobacillus* genera (Marotti et al., 2012). Prebiotic treatment is known to modulate host gene expression and metabolism as well (Everard et al., 2011). Dietary intervention using prebiotic inulin or oligofructose (FOS) alters the gut microflora composition by promoting the growth of beneficial bacteria such as *Bifidobacterium, Lactobacillus*, and *A. muciniphila* (Rossi et al., 2005; Choi and Shin, 2006). However, the mechanisms associating prebiotics and its beneficial effects have yet to be fully understood. Further studies are needed to examine the precise physiological roles of prebiotics on human bowel flora and in host immune function.

Xylooligosaccharide (XOS) is a recent prebiotic that can be incorporated into many food products (Aachary and Prapulla, 2011). Our lab previously reported that XOS, at the dose of 2.8 g/day, was well tolerated, and modified the gut bacterial composition in healthy people (Finegold et al., 2014). A consideration of the gut microbiota in the context of health benefits of XOS in Pre-DM is especially relevant since recent research has indicated a critical role of gut microbiota in the development of T2DM (Qin et al., 2012). The present study was designed to determine the effect of XOS supplementation on the gut microbiota in healthy and pre-DM individuals. Since the ultimate objective of this research is to explore the potential effects of XOS in the prevention of progression of Pre-DM to T2DM, we also evaluated the effects of XOS in the management of IGT, body composition, and inflammatory marker in Pre-DM subjects.

## Materials and methods

### Subjects

This was a double-blind, randomized, placebo-controlled study with 34 subjects who were recruited based on inclusion and exclusion criteria. The study population consisted of 16 healthy subjects (placebo: *n* = 9; XOS: *n* = 7) and 13 Pre-DM subjects (placebo: *n* = 6; XOS: *n* = 7).

### Ethics

The study was carried out in accordance with the guidelines of the Office for Protection of Research Subjects of the University of California, Los Angeles and the Institutional Review Board of the VA Greater LA Health Center. All subjects provided written informed consent before the study began.

### Study design

The enrollment criteria for healthy participants was fasting plasma glucose of 65–100 mg/dl, Participants in the Pre-DM study were selected based on the American Diabetes Association criteria for impaired fasting glucose (fasting plasma glucose of 100–125 mg/dl) and/or HgbA1c (5.7–6.4%) (American Diabetes Association, 2014). Over the span of 8 weeks, both healthy and Pre-DM subjects were randomly assigned to take daily a capsule supplement containing either 2 g XOS (2.8 grams of 70% XOS) or placebo. The XOS and placebo were provided by Life Bridge International (Riverside, CA). The XOS was manufactured by Shandong Longlive Bio-Technology Co., Ltd., China. The placebo capsules contained maltodextrin. The study consisted of three phases: a 2-week run-in phase, and an 8-week intervention phase.

### Stool collection

A total of two stools were collected from each subject: at baseline and week 8 of the intervention periods. Each time the entire stool specimen was obtained. The specimen was placed in a large, zip-lock freezer bag and all air was pushed out of the bag as the zip lock was closed. The specimen was delivered on ice to the UCLA Center for Human Nutrition within 24 h of collection where it was immediately stored at −20°C.

### Miseq sequencing

DNA from stool was extracted using a commercial extraction system (QIAamp Stool DNA Extraction Kit, Qiagen, Valencia, CA). The quality of the DNA samples was confirmed using a Bio-Rad Experion system (Bio-Rad Laboratories, CA, USA). The 16S rRNA gene V4 variable region PCR primers 515/806 with barcode on the forward primer were used in a 30 cycle PCR using the HotStarTaq Plus Master Mix Kit (Qiagen, USA) under the following conditions: 94°C for 3 min, followed by 28 cycles of 94°C for 30 s, 53°C for 40 s and 72°C for 1 min, after which a final elongation step at 72°C for 5 min was performed. After amplification, PCR products are checked in 2% agarose gel to determine the success of amplification and the relative intensity of bands. Sequencing was performed at MR DNA (www.mrdnalab.com, Shallowater, TX, USA) on a MiSeq following the manufacturer's guidelines. Sequence data were processed using a proprietary analysis pipeline (MR DNA, Shallowater, TX, USA). Operational taxonomic units (OTUs) were defined by clustering at 3% divergence (97% similarity). Final OTUs were taxonomically classified using BLASTn against a curated GreenGenes database (DeSantis et al., 2006).

### Body composition

Body composition was measured using the Tanita-BC418 bioelectrical impedance analyzer (Tanita Corp., Japan).

### Glucose tolerance test

On the test days at baseline and week 8, Pre-DM subjects remained in a fasting state for 2.5 h prior to the beginning of OGTT. The 75 g glucose cola was administrated immediately after the basal blood draw at 0 min. Subsequent blood samples were taken 30, 60, and 120 min afterwards. Serum samples were kept at −80°C. The insulin resistance index assessed by the homeostasis model (HOMA-IR) was calculated as follows (Allard et al., 2003): (fasting blood glucose [mmol/l] × fasting plasma insulin [μU/ml])/22.5.

### Blood biochemical analysis

Blood samples were collected and coded to protect patient confidentiality. Lipids, insulin, glucose, and satiety hormones were measured. Serum triglycerides were determined using a enzymatic method (Pointe Scientific, MI). Serum glucose was determined using Glucose Assay kit (Cayman Chemical Company, MI). Serum insulin, active GLP-1, leptin, pancreatic polypeptides (PP) and TNFα were determined using the MILLIPLEX map kit (EMD Millipore, Billerica, MA) and data were captured and processed using Luminext 200 with xPonent software.

### Statistics

Demographic data at baseline were analyzed and presented as mean ± SD and two sample *t*-tests were used for comparisons between the placebo and XOS groups. For analysis of serum variables, such as insulin, glucose, values of mean ± SE at baseline and week 8 were presented for all groups, and two sample *t*-test was used to compare the two groups at baseline and 8 weeks. For DNA sequencing analyses, Wilcoxon rank sum test was utilized to evaluate the differences between study groups. All tests are two sided and all analyses were conducted using SAS 9.3 (Statistical Analysis System, Cary, NC, 2008) and R (www.r-project.org) software.

## Results

### Subjects

The study population consisted of 16 healthy participants (4 men and 12 women) aged between 21 and 49 years, and 13 Pre-DM participants (9 men and 4 women) aged between 30 and 63. Participants did not report any adverse effects or symptoms with the XOS intervention at 2 g/day. Table 1 shows the baseline characteristics of the participants in the four groups. The composition of the fecal microbiome from all healthy participants and Pre-DM participants was analyzed by Miseq sequencing.

**Table 1:** **Baseline characteristics of the study participants**.

	**Healthy placebo (*n* = 9)**	**Healthy XOS (*n* = 7)**	**Pre-DM placebo (*n* = 6)**	**Pre-DM XOS (*n* = 7)**
Age, year	31.9 ± 6.9	31.7 ± 9.3	44.8 ± 11.2	55.0 ± 6.2
Weight, kg	65.4 ± 14.0	69.9 ± 13.0	94.9 ± 15.9	97.4 ± 22.7
BMI, kg/m^2^	23.4 ± 3.2	25.6 ± 3.2	33.6 ± 7.2	32.2 ± 4.2

*Values are presented as mean ± standard deviation (SD). For all characteristics, there were no significant differences between the placebo and XOS groups (all P > 0.05, based on independent sample t-tests). BMI, body mass index*.

### Gut microbial composition changes related to Pre-DM

**Figure 1:**
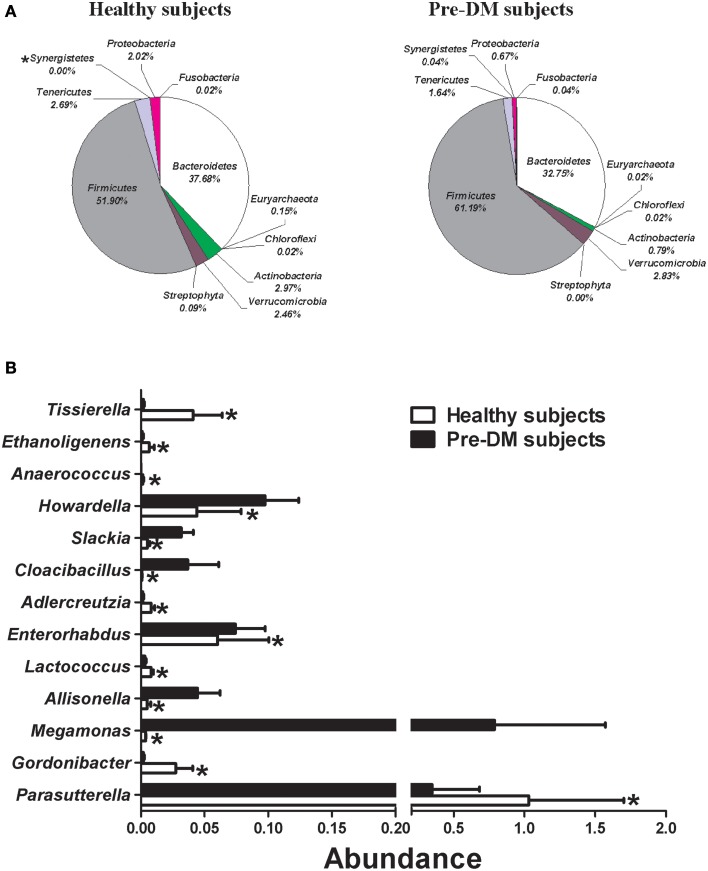
**Comparison of gut microbiota composition between healthy (*n* = 16) and Pre-DM (*n* = 13) subjects. (A)** Pie charts depict mean abundance (% of total) of the indicated phyla. **(B)** Bar graph of genera shows significant differences in abundance between healthy and Pre-DM subjects. Values are presented as mean ± standard error (SE) ^*^P ≤ 0.05.

Miseq sequencing was used to compare the gut microbial composition of baseline samples from 16 healthy and 13 Pre-DM subjects. The abundance (percentage of total sequences) of 1 phylum, 1 class, 3 families, 13 genera, and 22 species, was significantly different between healthy and Pre-DM (Supplementary Table 1). Composition of phyla in healthy and Pre-DM groups is displayed in Figure 1A. The abundance of infectious and T2DM related phylum *Synergistetes* (Baumgartner et al., 2012; Qin et al., 2012) was significantly higher in Pre-DM compared with healthy subjects (*P*≤0.05) (Figure 1A). In addition, 13 genera responded significantly in Pre-DM (*P*≤0.05) (Figure 1B). The abundances of *Allisonella, Cloacibacillus, Enterorhabdus, Howardella, Megamonas*, and *Slackia* were significantly higher, while *Adlercreutzia, Anaerococcus, Ethanoligenens, Gordonibacter, Lactococcus, Parasutterella*, and *Tissierella* were greatly reduced in Pre-DM compared with healthy subjects (Figure 1B).

### Effects of XOS supplementation on the gut microbiota in healthy and Pre-DM subjects

**Figure 2:**
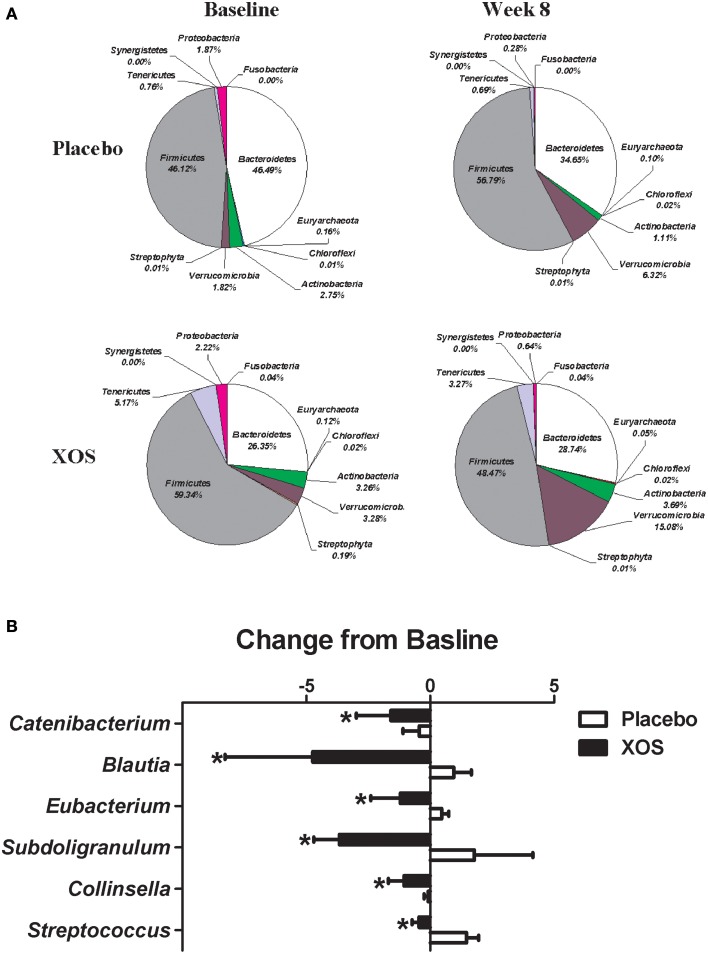
**Effects of XOS supplementation on gut microbiota in healthy subjects (*n* = 16). (A)** Pie charts display the mean abundance of indicated phyla of healthy subjects receiving placebo (*n* = 9) and XOS (*n* = 7) at baseline and week 8. **(B)** Bar graph of genera shows significant difference in abundance between placebo and XOS groups. Values are presented as mean ± standard error (SE) ^*^P ≤ 0.05.

**Figure 3:**
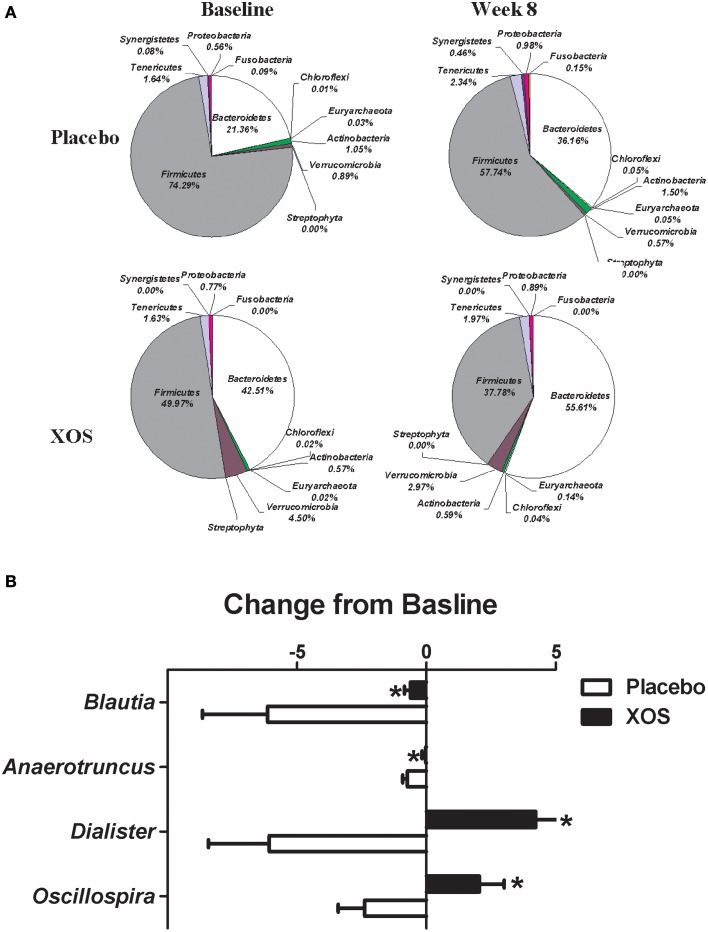
**Effects of XOS supplementation on gut microbiota in Pre-DM subjects (*n* = 13). (A)** Pie charts display the mean abundance of indicated phyla of Pre-DM subjects receiving placebo (*n* = 6) and XOS (*n* = 7) at baseline and week 8. **(B)** Bar graph of genera shows significant difference in abundance between placebo and XOS groups. Values are presented as mean ± standard error (SE) ^*^P ≤ 0.05.

The overall changes of bacterial composition with 8-week XOS intervention were assessed at the phylum and genus levels (Figures 2, 3). Significant XOS-induced changes were found for 1 phyla, 3 classes, 7 families, 23 genera, and 25 species in healthy subjects (Figure 2 and Supplementary Table 2), and 3 classes, 1 families, 7 genera, and 17 species in Pre-DM subjects (Figure 3 and Supplementary Table 3). The mean abundance of indicated phyla of healthy subjects in placebo and XOS groups at baseline and week 8 are displayed in Figure 2A. The phylum *Firmicutes* showed a 20% increase in abundance over 8 weeks in placebo groups of healthy subjects, while XOS intervention significantly reversed this increase (*P* ≤ 0.05) (Figure 2A). The mean abundance of *Verrucomicrobia* increased in XOS groups of healthy subjects as well. Among genera with an average abundance >1% in at least one group, six were significantly regulated by XOS. An increase of infectious disease related *Streptococcus* and *Subdoligranulum* in placebo groups was largely inhibited by XOS in healthy subjects (Figure 2B). The 8-week XOS intervention did not induce significant changes of gut microbiota at the phylum level in Pre-DM subjects (Figure 3A). At the genus level, *Blautia, Anaerotruncus, Dialister*, and *Oscillospira* were four abundant genera identified with significant XOS-induced changes in Pre-DM subjects. XOS diminished or reversed the magnitude of population decline in all four genera (Figure 3B).

### XOS supplementation reversed gut bacterial alterations associated with Pre-DM

**Figure 4:**
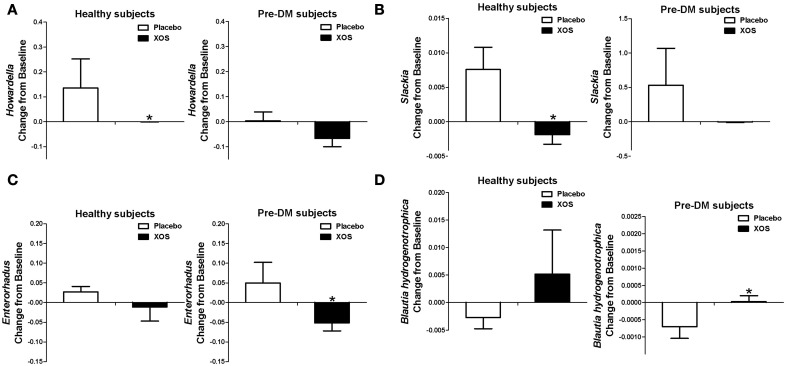
**XOS selectively regulated some of the Pre-DM associated bacterial taxa in healthy subjects (*n* = 16) or Pre-DM subjects (*n* = 13) during 8 weeks**. The abundances of Pre-DM associated *Howardella* **(A)**, *Slackia* **(B)**, and *Enterorhabdus* **(C)** were greatly reduced by XOS in healthy and Pre-DM subjects, respectively. **(D)** The abundance of healthy associated *Blautia hydrogenotrophica* was enhanced by XOS in healthy and Pre-DM subjects. Values are presented as mean ± standard error (SE) ^*^P ≤ 0.05.

Of the 40 Pre-DM associated bacterial taxa (Supplementary Table 1) identified in this study, the abundances of the *Enterorhabdus, Howardella*, and *Slackia* genera were elevated in Pre-DM. The 8-week XOS intervention significantly diminished or reversed the abundance increase of *Howardella* and *Slackia* observed in the placebo group of healthy subjects, as well as *Enterorhabdus* in Pre-DM subjects (Figures 4A–C). *B. hydrogenotrophica* was less abundant in Pre-DM subjects (Supplementary Table 1), but XOS intervention significantly reversed the decrease in *B. hydrogenotrophica* abundance observed in the placebo group of Pre-DM subjects (*P* ≤ 0.05) (Figure 4D).

### Effects of XOS on body composition, metabolic, and immunological markers in Pre-DM subjects

**Figure 5:**
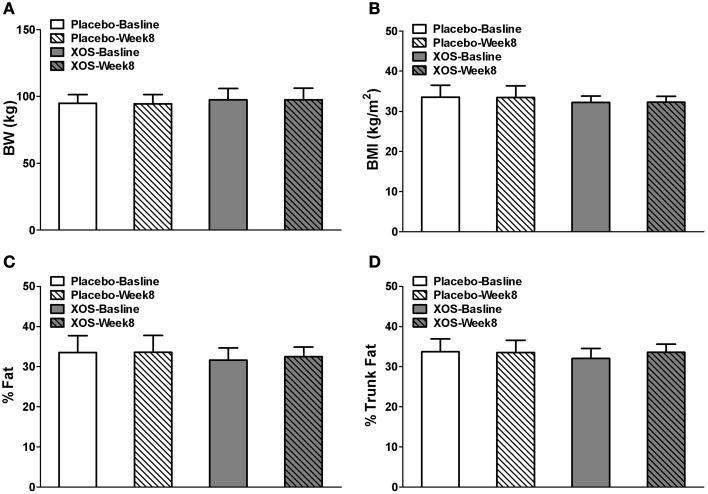
**Body weight (A), BMI (B), % Fat (C), and % Trunk fat (D) in Pre-DM subjects at baseline and after 8 weeks placebo (*n* = 6) or XOS (*n* = 7) treatment**. Data are means ± standard errors (SE).

**Figure 6:**
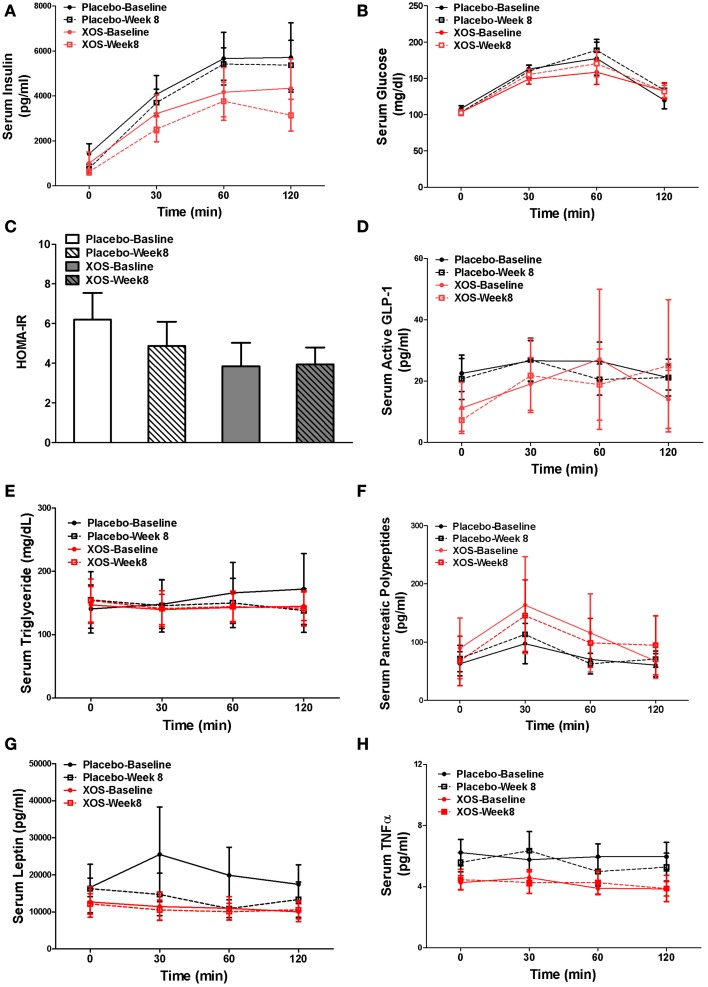
**Mean of parameters with SE at baseline and 8 weeks were compared between placebo- (*n* = 6) and XOS-treated (*n* = 7) group in Pre-DM subjects during the 120-min OGTT test. (A)** Serum Insulin. **(B)** Serum glucose. **(C)** HOMA-IR. **(D)** Serum active GLP-1. **(E)** Serum triglyceride. **(F)** Serum pancreatic polypeptides. **(G)** Serum leptin. **(H)** Serum TNF α. Values are presented as mean ± standard error (SE).

In Pre-DM subjects, body composition, blood tests, and oral glucose tolerance tests (OGTT) were done at baseline and after 8 weeks of XOS intervention. Body weight and indexes of overall adiposity such as BMI, % fat, and % trunk fat were not changed by 8-week XOS intervention (Figure 5). Despite significant inter-individual variations in insulin responses among Pre-DM subjects, OGTT 2-h insulin response showed a tendency to decrease with XOS intervention in Pre-DM (*P* = 0.13) (Figure 6A). No significant XOS-related differences were observed in serum glucose, HOMA-IR, active GLP-1, triglycerides, leptin, PP, or the inflammatory marker TNFα (Figures 6B–H).

## Discussion

Emerging evidence suggests that metabolic disorders including T2DM are associated with a pro-inflammatory state secondary to dysbiosis of gut bacterial flora (Larsen et al., 2010; Esteve et al., 2011; Musso et al., 2011). In addition, literature has documented the translocation of gut bacteria to blood and tissues in T2DM, and probiotic *Bifidobacterium* treatment prevents bacterial translocation and protects against T2DM (Cani et al., 2007; Amar et al., 2011). Together, these findings suggest that gut bacteria are an important modifier of T2DM.

The effects of oligosaccharides including XOS, FOS, and galactooligosaccharides (GOS) in the treatment of T2DM have gained interest. However, studies have shown inconsistent results. In T2DM patients, Yamashita et al. demonstrated that FOS at a dose of 8 g per day for 14 days resulted in a reduction of serum glucose, while Alles et al. showed that daily consumption of FOS at 15 g for 20 days had no effect on serum glucose level (Yamashita et al., 1984; Alles et al., 1999). Chan et al. showed that 4 g per day of XOS for 8 weeks was effective in reducing blood glucose and lipids in Taiwan T2DM patients (Sheu et al., 2008), and for 21 days benefited intestinal health and increased of *Bifidobacteria* in elderly subjects (Chung et al., 2007). Another study reported that both XOS and FOS dietary intervention reduced hyperglycaemia in diabetic rats (Gobinath et al., 2010). In addition, we found that 2 g per day of XOS for 8 weeks increased the *Bifidobacteria* abundance in healthy Americans without any gastrointestinal side effects (Finegold et al., 2014). Overall, a significant number of studies have shown oligosaccharides to be an effective option for lowering blood sugar in T2DM as well as improving intestinal health.

In the present study, both healthy and Pre-DM subjects were given 2 g per day of XOS for 8 weeks. Miseq sequencing was used to evaluate the potential of XOS in preventing the dysbiosis of gut microbiota during the development of T2DM. We found that XOS had a clear impact on gut microbiota in both healthy and Pre-DM groups, and resulted in dramatic shifts of several bacterial taxa associated with Pre-DM. Among them, *Dialister spp*. and *Slackia* are pro-inflammatory (Rocas and Siqueira, 2006; Kim et al., 2010), and were greatly reduced by XOS. Additionally, T2DM associated lactic acid bacteria *Enterococcus, Streptococcus*, and *Lactobacillus* (Remely et al., 2013) were also greatly reduced by XOS. The inhibitory effect of XOS on other opportunistic pathogens, such as *Clostridia, Streptococcaceae*, and *Subdoligranulum*, further supports that XOS can potentially promote an optimal gut microbiota profile, and consequently reduce the risk of T2DM.

Miseq sequencing data also revealed that gut microbial composition of healthy subjects at different taxonomic levels was different from Pre-DM subjects. Some of our findings are consistent with previous T2DM studies of gut microflora (Qin et al., 2012; Zhang et al., 2013; Finegold et al., 2014), substantiating the significant association of these changes with the progression of T2DM (Supplementary Table 1). Studies showed that the *Megamonas* OTU was most enriched in Pre-DM, compared to healthy or T2DM individuals (Qin et al., 2012; Zhang et al., 2013). The abundance of *Megamonas* in our study was 200-fold higher in Pre-DM than healthy subjects. We also found that the abundance of the phylum *Synergistetes* in Pre-DM was about 50 fold higher compared with healthy subjects. The *Synergistetes* appear to be more numerous in individuals with oral-related diseases as well as gut and soft tissue infections (Vartoukian et al., 2007). Another two infectious or metabolic disease related bacteria *Eubacteriaceae* (Plieskatt et al., 2013) and *Slackia* (Kim et al., 2010), were more abundant in Pre-DM individuals. The Pre-DM associated enrichment of infectious bacteria and the appearance of oral bacteria in the gut suggest that the host immune system may lose control over these opportunistic pathogens during the development of T2DM.

Both animal and clinical studies have shown that XOS supplementation greatly increases the *Bifidobacterium* population (Campbell et al., 1997; Chung et al., 2007; Finegold et al., 2014). We previously showed that an increase of *Bifidobacterium* abundance was detectable only with an *in vitro* culture method, and not pyrosequencing (Finegold et al., 2014). Using Miseq sequencing alone, the abundances of the *Bifidobacterium* genus, as well as the *Bifidobacterium longum, Bifidobacterium bifidum*, and *Bifidobacterium adolescentis* species were not significantly increased by XOS in healthy subjects (Supplementary Table 4). However, we found that XOS largely inhibited bacterial taxa related to infectious and metabolic disease, such as family *Streptococcaceae*, class *Clostridia*, and genera *Subdoligranulum, Gordonibacter*, and *Streptococcus* in healthy subjects (Supplementary Table 1). Furthermore, in healthy subjects, XOS greatly reduced the abundance of bacteria related to obesity or T2DM, including phylum *Firmicutes* and genera *Subdoligranulum* and *Bacilli* (Remely et al., 2013; Zhang et al., 2013). In Pre-DM subjects, XOS diminished or reversed the magnitude of population decline in about 70% bacterial taxa identified with a significant change from its baseline levels between treatment groups (Supplementary Table 2). The family *Veillonellaceae* and genera *Oscillospira* and *Dialister* exhibited population declines in the placebo group, but demonstrated large increases in abundance in the XOS group. Abnormally low levels of *Veillonellaceae* and *Dialister* have been described in autistic children (Kang et al., 2013) and patients of Crohn's disease (Joossens et al., 2011). Dietary whole grain intervention (Martinez et al., 2013) and corn fiber (Hooda et al., 2012) increased the *Dialister* and *Veillonellaceae* abundance. The genus *Oscillospira* has been associated with lean BMI (Tims et al., 2013). The inhibition of *Firmicutes* and increase of *Oscillospira* abundance suggest a potential role of XOS in weight control.

To the best of our knowledge, this is the first clinical study evaluating the effects of daily treatment with 2 g of XOS on glucose tolerance and insulin resistance in Pre-DM adults. In our experience, a dose of 2 g does not cause any gastrointestinal side effects (Finegold et al., 2014). Eight weeks of XOS supplementation tended to increase insulin sensitivity by lowering OGTT 2-h insulin response (*P* = 0.11), while no significant improvement of Pre-DM subjects' metabolic situation was observed, using the parameters of body composition, serum glucose, triglyceride, satiety hormones and inflammation marker TNFα. The TNFα levels (4.91 ± 1.85 pg/ml) of Pre-DM subjects in our study are normal, slightly lower than the reported TNFα (~15–20 pg/ml) of eastern Indian Pre-DM population (Dutta et al., 2013) and much lower than T2DM (range from 87 to 112 pg/ml) (Goyal et al., 2012). Since we enrolled pre-DM subjects with impaired glucose tolerance (IGT) and without any other medical conditions we possibly did not observe elevated TNFα level. Pre-DM is a dynamic intermediate stage in the progression to T2DM, therefore it is very likely, subjects that met the selection criteria for Pre-DM are at different stages even though they are all classified as Pre-DM. Besides, studies also suggest a connection between TNFα gene polymorphism, its blood levels and the tendency to progression from Pre-DM to T2DM (Dutta et al., 2013). Therefore, more studies are needed to improve our understanding of the relationship between TNAα, Pre-DM staging, and T2DM progression. Our results do not agree with Chan et al. study in T2DM. However, this discrepancy could be explained by difference in XOS dose, study population and disease stages. It is possible that 2 g per day of XOS may have been too low to induce a difference in glucose tolerance. However, we observed a trend of increased insulin sensitivity by lowering OGTT 2-h insulin response at this dosage. Pre-DM is a strong risk factor for the development of T2DM, and the regulation of glucose metabolism and insulin sensitivity could be really dynamic during this stage. We think future clinical study with large sample size will be needed to confirm the benefits of XOS in Pre-DM.

In conclusion, XOS significantly modified gut microbiota in both healthy and Pre-DM subjects, and resulted in dramatic shifts of 4 bacterial taxa associated with Pre-DM. Future studies with larger sample size are needed to study the metabolic impact of XOS and understand the connection between XOS-mediated gut microbiota changes and the pathogenesis of T2DM.

## Source of support

Supported by departmental funds from the Center for Human Nutrition, Department of Medicine, David Geffen School of Medicine, University of California, Los Angeles.

### Conflict of interest statement

The authors declare that the research was conducted in the absence of any commercial or financial relationships that could be construed as a potential conflict of interest.

## Abbreviations

XOS: xylooligosaccharide
Pre-DM: prediabetic
BMI: body mass index
T2DM: Type 2 Diabetes Mellitus
FOS: fructooligosaccharides
GOS: galactooligosaccharides
OGTT: oral glucose tolerance test

## References

[B1] Aachary A. A. Prapulla S. G. (2011). Xylooligosaccharides (XOS) as an emerging prebiotic: microbial synthesis, utilization, structural characterization, bioactive properties, and applications. Compr. Rev. Food Sci. Food Saf. 10, 2–16. 10.1111/j.1541-4337.2010.00135.x

[B2] Allard P. Delvin E. E. Paradis G. Hanley J. A. O'Loughlin J. Lavallée C.. (2003). Distribution of fasting plasma insulin, free fatty acids, and glucose concentrations and of homeostasis model assessment of insulin resistance in a representative sample of Quebec children and adolescents. Clin. Chem. 49, 644–649. 10.1373/49.4.644 12651818

[B3] Alles M. S. de Roos N. M. Bakx J. C. van de Lisdonk E. Zock P. L. Hautvast G. A. (1999). Consumption of fructooligosaccharides does not favorably affect blood glucose and serum lipid concentrations in patients with type 2 diabetes. Am. J. Clin. Nutr. 69, 64–69. 9925124 10.1093/ajcn/69.1.64

[B4] Amar J. Chabo C. Waget A. Klopp P. Vachoux C. Bermúdez-Humarán L. G.. (2011). Intestinal mucosal adherence and translocation of commensal bacteria at the early onset of type 2 diabetes: molecular mechanisms and probiotic treatment. EMBO Mol. Med. 3, 559–572. 10.1002/emmm.201100159 21735552 PMC3265717

[B5] American Diabetes Association (2014). Standards of medical care in diabetes–2014. Diabetes Care 37(Suppl. 1), S14–S80. 10.2337/dc14-S014 24357209

[B6] Baumgartner A. Thurnheer T. Lüthi-Schaller H. Gmür R. Belibasakis G. N. (2012). The phylum Synergistetes in gingivitis and necrotizing ulcerative gingivitis. J. Med. Microbiol. 61(Pt 11), 1600–1609. 10.1099/jmm.0.047456-0 22878253

[B7] Campbell J. M. Fahey G. C. Jr. Wolf B. W. (1997). Selected indigestible oligosaccharides affect large bowel mass, cecal and fecal short-chain fatty acids, pH and microflora in rats. J. Nutr. 127, 130–136. 9040556 10.1093/jn/127.1.130

[B8] Cani P. D. Neyrinck A. M. Fava F. Knauf C. Burcelin R. G. Tuohy K. M.. (2007). Selective increases of bifidobacteria in gut microflora improve high-fat-diet-induced diabetes in mice through a mechanism associated with endotoxaemia. Diabetologia 50, 2374–2383. 10.1007/s00125-007-0791-0 17823788

[B9] Chassaing B. Gewirtz A. T. (2014). Gut microbiota, low-grade inflammation, and metabolic syndrome. Toxicol. Pathol. 42, 49–53. 10.1177/0192623313508481 24285672

[B10] Choi N. Shin H. S. (2006). Effect of oligosaccharides and inulin on the growth and viability of bifidobacteria in skim milk. Food Sci. Biotechnol. 15, 543–548.

[B11] Chung Y. C. Hsu C. K. Ko C. Y. Chan Y. C. (2007). Dietary intake of xylooligosaccharides improves the intestinal microbiota, fecal moisture, and pH value in the elderly. Nutr. Res. 27, 756–761. 10.1016/j.nutres.2007.09.014

[B12] DeSantis T. Z. Hugenholtz P. Larsen N. Rojas M. Brodie E. L. Keller K.. (2006). Greengenes, a chimera-checked 16S rRNA gene database and workbench compatible with ARB. Appl. Environ. Microbiol. 72, 5069–5072. 10.1128/AEM.03006-05 16820507 PMC1489311

[B13] Dutta D. Choudhuri S. Mondal S. A. Maisnam I. Reza A. H. Ghosh S.. (2013). Tumor necrosis factor alpha -238G/A (rs 361525) gene polymorphism predicts progression to type-2 diabetes in an Eastern Indian population with prediabetes. Diab. Res. Clin. Pract. 99, e37–e41. 10.1016/j.diabres.2012.12.007 23298660

[B14] Esteve E. Ricart W. Fernández-Real J. M. (2011). Gut microbiota interactions with obesity, insulin resistance and type 2 diabetes: did gut microbiote co-evolve with insulin resistance? Curr. Opin. Clin. Nutr. Metab. Care 14, 483–490. 10.1097/mco.0b013e328348c06d 21681087

[B15] Everard A. Belzer C. Geurts L. Ouwerkerk J. P. Druart C. Bindels L. B.. (2013). Cross-talk between *Akkermansia muciniphila* and intestinal epithelium controls diet-induced obesity. Proc. Natl. Acad. Sci. U.S.A. 110, 9066–9071. 10.1073/pnas.1219451110 23671105 PMC3670398

[B16] Everard A. Lazarevic V. Derrien M. Girard M. Muccioli G. G. Neyrinck A. M.. (2011). Responses of gut microbiota and glucose and lipid metabolism to prebiotics in genetic obese and diet-induced leptin-resistant mice. Diabetes 60, 2775–2786. 10.2337/db11-0227 21933985 PMC3198091

[B17] Finegold S. M. Li Z. Summanen P. H. Downes J. Thames G. Corbett K.. (2014). Xylooligosaccharide increases bifidobacteria but not lactobacilli in human gut microbiota. Food Funct. 5, 436–445. 10.1039/c3fo60348b 24513849

[B18] Gobinath D. Madhu A. N. Prashant G. Srinivasan K. Prapulla S. G. (2010). Beneficial effect of xylo-oligosaccharides and fructo-oligosaccharides in streptozotocin-induced diabetic rats. Br. J. Nutr. 104, 40–47. 10.1017/S0007114510000243 20187988

[B19] Goyal R. Faizy A. F. Siddiqui S. S. Singhai M. (2012). Evaluation of TNF-alpha and IL-6 levels in obese and non-obese diabetics: pre- and postinsulin effects. N. Am. J. Med. Sci. 4, 180–184. 10.4103/1947-2714.94944 22536561 PMC3334258

[B20] Hooda S. Boler B. M. V. Serao M. C. R. Brulc J. M. Staeger M. A. Boileau T. W.. (2012). 454 pyrosequencing reveals a shift in fecal microbiota of healthy adult men consuming polydextrose or soluble corn fiber. J. Nutr. 142, 1259–1265. 10.3945/jn.112.158766 22649263

[B21] International Scientific Association for Probiotics Prebiotics. (2004). Probiotics, prebiotics, and new foods. Proceedings of the 2nd annual meeting. Rome, Italy, September 7–9, 2003. J. Clin. Gastroenterol. 38(6 Suppl.), S60–S129. 15220659

[B22] Joossens M. Huys G. Cnockaert M. De Preter V. Verbeke K. Rutgeerts P.. (2011). Dysbiosis of the faecal microbiota in patients with Crohn's disease and their unaffected relatives. Gut 60, 631–637. 10.1136/gut.2010.223263 21209126

[B23] Kang D. W. Park J. G. Ilhan Z. E. Wallstrom G. Labaer J. Adams J. B.. (2013). Reduced incidence of Prevotella and other fermenters in intestinal microflora of autistic children. PLoS ONE 8:e68322. 10.1371/journal.pone.0068322 23844187 PMC3700858

[B24] Kim K. S. Rowlinson M. C. Bennion R. Liu C. Talan D. Summanen P.. (2010). Characterization of Slackia exigua isolated from human wound infections, including abscesses of intestinal origin. J. Clin. Microbiol. 48, 1070–1075. 10.1128/JCM.01576-09 20107092 PMC2849566

[B25] Larsen N. Vogensen F. K. van den Berg F. W. Nielsen D. S. Andreasen A. S. Pedersen B. K.. (2010). Gut microbiota in human adults with type 2 diabetes differs from non-diabetic adults. PLoS ONE 5:e9085. 10.1371/journal.pone.0009085 20140211 PMC2816710

[B26] Marotti I. Bregola V. Aloisio I. Di Gioia D. Bosi S. Di Silvestro R.. (2012). Prebiotic effect of soluble fibres from modern and old durum-type wheat varieties on Lactobacillus and Bifidobacterium strains. J. Sci. Food Agric. 92, 2133–2140. 10.1002/jsfa.5597 22298124

[B27] Martínez I. Lattimer J. M. Hubach K. L. Case J. A. Yang J. Weber C. G.. (2013). Gut microbiome composition is linked to whole grain-induced immunological improvements. ISME J. 7, 269–280. 10.1038/ismej.2012.104 23038174 PMC3554403

[B28] Musso G. Gambino R. Cassader M. (2011). Interactions between gut microbiota and host metabolism predisposing to obesity and diabetes. Annu. Rev. Med. 62, 361–80. 10.1146/annurev-med-012510-175505 21226616

[B29] Plieskatt J. L. Deenonpoe R. Mulvenna J. P. Krause L. Sripa B. Bethony J. M.. (2013). Infection with the carcinogenic liver fluke Opisthorchis viverrini modifies intestinal and biliary microbiome. Faseb J. 27, 4572–84. 10.1096/fj.13-232751 23925654 PMC3804743

[B30] Portero McLellan K. C. Wyne K. Villagomez E. T. Hsueh W. A. (2014). Therapeutic interventions to reduce the risk of progression from prediabetes to type 2 diabetes mellitus. Ther. Clin. Risk Manag. 10, 173–188. 10.2147/TCRM.S39564 24672242 PMC3964168

[B31] Qin J. Li Y. Cai Z. Li S. Zhu J. Zhang F.. (2012). A metagenome-wide association study of gut microbiota in type 2 diabetes. Nature 490, 55–60. 10.1038/nature11450 23023125

[B32] Remely M. Dworzak S. Hippe B. Zwielehner J. Aumüller E. Brath H. (2013). Abundance and diversity of microbiota in type 2 diabetes and obesity. J. Diab. Metab. 4:253 10.4172/2155-6156.1000253

[B33] Rocas I. N. Siqueira J. F. Jr. (2006). Characterization of Dialister species in infected root canals. J. Endod. 32, 1057–1061. 10.1016/j.joen.2006.04.010 17055906

[B34] Rossi M. Corradini C. Amaretti A. Nicolini M. Pompei A. Zanoni S.. (2005). Fermentation of fructooligosaccharides and inulin by bifidobacteria: a comparative study of pure and fecal cultures. Appl. Environ. Microbiol. 71, 6150–6158. 10.1128/AEM.71.10.6150-6158.2005 16204533 PMC1265942

[B35] Shen J. Obin M. S. Zhao L. (2013). The gut microbiota, obesity and insulin resistance. Mol. Aspects Med. 34, 39–58. 10.1016/j.mam.2012.11.001 23159341

[B36] Sheu W. H. Lee I. T. Chen W. Chan Y. C. (2008). Effects of xylooligosaccharides in type 2 diabetes mellitus. J. Nutr. Sci. Vitaminol. (Tokyo) 54, 396–401. 10.3177/jnsv.54.396 19001772

[B37] Tims S. Derom C. Jonkers D. M. Vlietinck R. Saris W. H. Kleerebezem M.. (2013). Microbiota conservation and BMI signatures in adult monozygotic twins. ISME J. 7, 707–717. 10.1038/ismej.2012.146 23190729 PMC3603393

[B38] Vartoukian S. R. Palmer R. M. Wade W. G. (2007). The division “Synergistes”. Anaerobe 13, 99–106. 10.1016/j.anaerobe.2007.05.004 17631395

[B39] Yamashita K. Kawai K. Itakura M. (1984). Effects of fructo-oligosaccharides on blood-glucose and serum-lipids in diabetic subjects. Nutr. Res. 4, 961–966. 10.1016/S0271-5317(84)80075-5

[B40] Zhang X. Shen D. Fang Z. Jie Z. Qiu X. Zhang C.. (2013). Human gut microbiota changes reveal the progression of glucose intolerance. PLoS ONE 8:e71108. 10.1371/journal.pone.0071108 24013136 PMC3754967

