# Supplementary material for: Xylooligosaccharide supplementation alters gut bacteria in both healthy and prediabetic adults: a pilot study
Source: Front Physiol. 2015 Aug 7;6:216. doi: 10.3389/fphys.2015.00216 (PMC4528259; doi:10.3389/fphys.2015.00216)
Supplement: Supplementary file 1 [file DataSheet1.DOC]

**Supplementary Table 1: The list of significant different bacterial taxa (% total microbiota) between healthy and Pre-DM subjects**

| **Taxonomy assignment** | | ***P value*** | **Abundance** | | **Enrichment** | | **Reference** |
| --- | --- | --- | --- | --- | --- | --- | --- |
| **Name** | **Level** |  | **Health** | **Pre-DM** | 0=H, 1= Pre-DM | Published (0=H, 1=infectious or metabolic disease) |  |
| *Synergistetes* | phylum | 0.03 | 0.0007 ± 0.0009 | 0.0367 ± 0.0878 | 1 | 1 |  |
| *Synergistia* | class | 0.03 | 0.0007 ± 0.0059 | 0.0367 ± 0.7557 | 1 | 1 | [1] |
| *Alcaligenaceae* | family | 0.02 | 1.4700 ± 0.3957 | 0.3700 ± 0.9070 | 0 | 1 |  |
| *Synergistaceae* | family | 0.03 | 0.0007 ± 0.0059 | 0.0367 ± 0.7557 | 1 | 1 | [1] |
| *Eubacteriaceae* | family | 0.05 | 0.0000 ± 0.0002 | 0.0006 ± 0.0049 | 1 | 0 |  |
| *Adlercreutzia* | genus | 0.02 | 0.0078 ± 0.0119 | 0.0015 ± 0.0028 | 0 |  |  |
| *Allisonella* | genus | 0.05 | 0.0049 ± 0.0107 | 0.0444 ± 0.0643 | 1 | 1 |  |
| *Anaerococcus* | genus | 0.03 | 0.0014 ± 0.0029 | 0.0000 ± 0.0000 | 0 | 1 |  |
| *Cloacibacillus* | genus | 0.03 | 0.0007 ± 0.0009 | 0.0367 ± 0.0878 | 1 |  |  |
| *Enterorhabdus* | genus | 0.03 | 0.0600 ± 0.1604 | 0.0700 ± 0.0841 | 1 |  |  |
| *Ethanoligenens* | genus | 0.03 | 0.0063 ± 0.0161 | 0.0010 ± 0.0035 | 0 |  |  |
| *Gordonibacter* | genus | 0.03 | 0.0300 ± 0.0529 | 0.0020 ± 0.0026 | 0 |  |  |
| *Howardella* | genus | 0.03 | 0.0400 ± 0.1391 | 0.1000 ± 0.0957 | 1 |  |  |
| *Lactococcus* | genus | 0.04 | 0.0080 ± 0.0080 | 0.0030 ± 0.0033 | 0 |  |  |
| *Megamonas* | genus | 0.02 | 0.0040 ± 0.0015 | 0.7880 ± 2.8216 | 1 | 1 |  |
| *Parasutterella* | genus | 0.05 | 1.0300 ± 2.6894 | 0.3500 ± 1.2089 | 0 | 1 |  |
| *Slackia* | genus | 0.01 | 0.0050 ± 0.0075 | 0.0320 ± 0.0338 | 1 | 1 |  |
| *Tissierella* | genus | 0.03 | 0.0410 ± 0.0919 | 0.0020 ± 0.0023 | 0 |  |  |
| *Blautia hydrogenotrophica* | species | 0.01 | 0.0061 ± 0.0158 | 0.0005 ± 0.0006 | 0 | 1(0) |  |
| *Slackia* | species | 0.01 | 0.0051 ± 0.0121 | 0.0317 ± 0.8920 | 1 |  |  |
| *Bacteroides fragilis* | species | 0.02 | 0.0400 ± 0.1400 | 0.0071 ± 0.0351 | 0 | 1 |  |
| *Adlercreutzia equolifaciens* | species | 0.02 | 0.0078 ± 0.0055 | 0.0015 ± 0.0020 | 0 |  |  |
| *Megamonas* | species | 0.02 | 0.0036 ± 0.0019 | 0.7882 ± 0.2047 | 1 |  |  |
| *Dialister human gut* | species | 0.02 | 0.6438 ± 2.6885 | 5.3053 ± 7.5970 | 1 |  |  |
| *Bacteroides caccae* | species | 0.03 | 1.7381 ± 1.7730 | 0.5108 ± 2.5753 | 0 | 1 |  |
| *Cloacibacillus* | species | 0.03 | 0.0007 ± 0.0059 | 0.0367 ± 0.7557 | 1 |  |  |
| *Dialister* | species | 0.03 | 0.0002 ± 0.0009 | 0.0027 ± 0.0038 | 1 |  |  |
| *Lactobacillus fermentum* | species | 0.03 | 0.0001 ± 0.0005 | 0.0079 ± 0.0050 | 1 |  |  |
| *Gordonibacter* | species | 0.03 | 0.0274 ± 0.2691 | 0.0019 ± 0.0088 | 0 |  |  |
| *Enterorhabdus* | species | 0.03 | 0.0602 ± 0.1843 | 0.0742 ± 0.1124 | 1 |  |  |
| *Eubacterium biforme* | species | 0.03 | 0.9294 ± 0.8074 | 1.2279 ± 3.8086 | 1 | 0(1) |  |
| *Anaerococcus hydrogenalis* | species | 0.03 | 0.0014 ± 0.0036 | 0.0000 ± 0.0018 | 0 |  |  |
| *Howardella ureilytica* | species | 0.03 | 0.0439 ± 0.4022 | 0.0973 ± 0.1068 | 1 |  |  |
| *Tissierella praeacuta* | species | 0.03 | 0.0409 ± 0.0813 | 0.0020 ± 0.0084 | 0 |  |  |
| *Ethanoligenens harbinense* | species | 0.03 | 0.0063 ± 0.0027 | 0.0010 ± 0.0018 | 0 | 1(0) |  |
| *Prevotella buccalis* | species | 0.04 | 0.0042 ± 0.0030 | 0.0002 ± 0.0004 | 0 |  |  |
| *Bacteroides massiliensis* | species | 0.04 | 0.3407 ± 1.7837 | 1.6898 ± 1.7143 | 1 |  |  |
| *Costridium sulfatireducens* | species | 0.05 | 0.0037 ± 0.0220 | 0.0137 ± 0.0072 | 1 |  |  |
| *Allisonella* | species | 0.05 | 0.0049 ± 0.0195 | 0.0444 ± 0.0794 | 1 |  |  |
| *Parasutterella* | species | 0.05 | 1.0298 ± 0.4068 | 0.3462 ± 0.9170 | 0 |  |  |

**Supplementary table 2: The list of bacterial taxa with significant differences in the mean change from baseline in healthy subjects**

| **Taxonomic assignment** | | ***p*-value** | **Abundance** | | | |
| --- | --- | --- | --- | --- | --- | --- |
| **Name** | **level** |  | **Placebo W0** | **Placebo W8** | **XOS W0** | **XOS W8** |
| *Firmicutes* | phylum | 0.01 | 59.3424 ± 12.5284 | 48.4677 ± 15.6761 | 46.1050 ± 19.1831 | 56.794 ± 9.4730 |
| *Bacilli* | class | 0.00 | 1.0773 ± 1.2186 | 2.6777 ± 2.6050 | 1.0127 ± 0.8005 | 0.3620 ± 0.2045 |
| *Coriobacteria* | class | 0.01 | 0.1521 ± 0.1225 | 0.2745 ± 0.4020 | 0.3993 ± 0.5131 | 0.2691 ± 0.4701 |
| *Clostridia* | class | 0.02 | 42.4084 ± 19.1685 | 52.1093 ± 10.1511 | 55.8892 ± 12.2423 | 47.4382 ± 15.8970 |
| *Streptococcaceae* | family | 0.01 | 0.9509 ± 1.1007 | 2.4175 ± 2.4025 | 0.6505 ± 0.6770 | 0.1963 ± 0.1017 |
| *Lactobacillaceae* | family | 0.01 | 0.1109 ± 0.1152 | 0.2264 ± 0.2451 | 0.1422 ± 0.0918 | 0.1055 ± 0.1260 |
| *Coriobacteriaceae* | family | 0.01 | 0.4115 ± 0.4947 | 0.4731 ± 0.664 | 1.9058 ± 1.8613 | 0.7198 ± 0.9636 |
| *Clostridiales x ince* | family | 0.03 | 0.0190 ± 0.0328 | 0.0165 ± 0.0171 | 0.1993 ± 0.4001 | 0.0962 ± 0.2305 |
| *Enterococcaceae* | family | 0.03 | 0.0021 ± 0.0022 | 0.0093 ± 0.0160 | 0.0228 ± 0.0270 | 0.0035 ± 0.0036 |
| *Gemellaceae* | family | 0.04 | 0.0015 ± 0.0032 | 0.0020 ± 0.0030 | 0.0123 ± 0.0136 | 0.0025 ± 0.0032 |
| *Dehalobacteriaceae* | family | 0.05 | 0.0079 ± 0.0103 | 0.0238 ± 0.0287 | 0.0041 ± 0.0085 | 0.0009 ± 0.0009 |
| *Streptococcus* | genus | 0.01 | 0.9452 ± 1.0939 | 2.4075 ± 2.3920 | 0.6399 ± 0.6768 | 0.1877 ± 0.1008 |
| *Lactobacillus* | genus | 0.01 | 0.1109 ± 0.1152 | 0.2264 ± 0.2451 | 0.1422 ± 0.0918 | 0.1055 ± 0.1260 |
| *Bryantella* | genus | 0.01 | 0.0009 ± 0.0010 | 0.0013 ± 0.0007 | 0.0034 ± 0.0023 | 0.0019 ± 0.0013 |
| *Collinsella* | genus | 0.02 | 0.3515 ± 0.4620 | 0.2670 ± 0.3046 | 1.5619 ± 1.6853 | 0.5094 ± 0.8478 |
| *Subdoligranulum* | genus | 0.02 | 5.2840 ± 6.8168 | 7.0640 ± 8.7096 | 9.5540 ± 4.7340 | 5.8916 ± 2.5767 |
| *Gordonibacter* | genus | 0.02 | 0.0265 ± 0.0644 | 0.1328 ± 0.3586 | 0.0284 ± 0.0383 | 0.0136 ± 0.0151 |
| *Olsenella* | genus | 0.02 | 0.0018 ± 0.0028 | 0.0054 ± 0.0075 | 0.0255 ± 0.0665 | 0.0054 ± 0.0142 |
| *Enterococcus* | genus | 0.02 | 0.0021 ± 0.0022 | 0.0093 ± 0.0161 | 0.0228 ± 0.0270 | 0.0035 ± 0.0036 |
| *Eubacterium* | genus | 0.02 | 0.7513 ± 0.6327 | 1.2151 ± 1.1613 | 1.8708 ± 3.7144 | 0.6631 ± 1.1365 |
| *Parvimonas* | genus | 0.03 | 0.0010 ± 0.0019 | 0.0019 ± 0.0019 | 0.0315 ± 0.0658 | 0.0023 ± 0.0032 |
| *Slackia* | genus | 0.03 | 0.0064 ± 0.0094 | 0.0140 ± 0.0141 | 0.0034 ± 0.0038 | 0.0015 ± 0.0010 |
| *Papillibacter* | genus | 0.04 | 0.0164 ± 0.0167 | 0.0299 ± 0.0290 | 0.0147 ± 0.0133 | 0.0055 ± 0.0057 |
| *Moryella* | genus | 0.04 | 0.0341 ± 0.0236 | 0.0510 ± 0.0567 | 0.0962 ± 0.0994 | 0.0387 ± 0.0221 |
| *Gemella* | genus | 0.04 | 0.0015 ± 0.0032 | 0.0020 ± 0.0029 | 0.0123 ± 0.0136 | 0.0025 ± 0.0032 |
| *Anaerosporobacter* | genus | 0.04 | 0.0012 ± 0.0025 | 0.0022 ± 0.0045 | 0.0002 ± 0.0005 | 0.0000 + 0.0000 |
| *Finegoldia* | genus | 0.04 | 0.0004 ± 0.0013 | 0.0003 ± 0.0005 | 0.0070 ± 0.0126 | 0.0019 ± 0.0039 |
| *Anaerofilum* | genus | 0.05 | 0.0033 ± 0.0034 | 0.0078 ± 0.0100 | 0.0023 ± 0.0034 | 0.0021 ± 0.0038 |
| *Blautia* | genus | 0.05 | 2.7150 ± 1.9004 | 3.6683 ± 1.7440 | 11.0699 ± 9.8454 | 6.3318 ± 6.9098 |
| *Sporobacter* | genus | 0.05 | 0.0173 ± 0.0083 | 0.0311 ± 0.0263 | 0.0071 ± 0.0151 | 0.0024 ± 0.0033 |
| *Anaerococcus* | genus | 0.05 | 0.0008 ± 0.0020 | 0.0029 ± 0.0047 | 0.0023 ± 0.0038 | 0.0007 ± 0.0007 |
| *Dehalobacterium* | genus | 0.05 | 0.0079 ± 0.0103 | 0.0238 ± 0.0287 | 0.0041 ± 0.0085 | 0.0009 ± 0.0009 |
| *Catenibacterium* | genus | 0.05 | 2.3781 ± 3.3400 | 1.9436 ± 2.8891 | 2.1627 ± 4.2146 | 0.5781 ± 0.9075 |
| *Howardella* | genus | 0.05 | 0.0764 ± 0.1832 | 0.2121 ± 0.5307 | 0.0023 ± 0.0018 | 0.0021 ± 0.0020 |
| *Collinsella aerofaciens* | species | 0.01 | 0.2365 ± 0.4741 | 0.1636 ± 0.2708 | 1.4394 ± 1.6017 | 0.4162 ± 0.8320 |
| *Streptococcus salivarius* | species | 0.01 | 0.3322 + 0.6786 | 0.4855 + 1.3408 | 0.2560 + 0.4095 | 0.0456 + 0.0416 |
| *Bryantella formatexigens* | species | 0.01 | 0.0009 ± 0.0010 | 0.0013 ± 0.0007 | 0.0034 ± 0.0023 | 0.0019 ± 0.0013 |
| *Alistipes indistinctus* | species | 0.02 | 0.0294 ± 0.0479 | 0.0194 ± 0.0339 | 0.0409 ± 0.0697 | 0.1781 ± 0.4419 |
| *Subdoligranulum* | species | 0.02 | 5.2840 ± 6.8168 | 7.0640 ± 8.7096 | 9.5540 ± 4.7341 | 5.8916 ± 2.5767 |
| *Gordonibacter* | species | 0.02 | 0.0265 ± 0.0644 | 0.1328 ± 0.3586 | 0.0284 ± 0.0383 | 0.0136 ± 0.0151 |
| *Olsenella* | species | 0.02 | 0.0018 ± 0.0028 | 0.0054 ± 0.0075 | 0.0255 ± 0.0665 | 0.0054 ± 0.0142 |
| *Slackia* | species | 0.02 | 0.0064 ± 0.0094 | 0.0140 ± 0.0141 | 0.0034 ± 0.0038 | 0.0015 ± 0.0010 |
| *Enterococcus ratti* | species | 0.02 | 0.0021 + 0.0022 | 0.0093 + 0.0160 | 0.0228 + 0.0270 | 0.0035 + 0.0036 |
| *Streptococcus australis* | species | 0.02 | 0.2290 ± 0.4583 | 0.2308 ± 0.6083 | 0.1788 ± 0.2735 | 0.0403 ± 0.0258 |
| *Parvimonas micra* | species | 0.03 | 0.0010 ± 0.0019 | 0.0019 ± 0.0019 | 0.0315 ± 0.0658 | 0.0023 ± 0.0032 |
| *Bacteroides eggerthii* | species | 0.03 | 0.7332 ± 1.6328 | 0.5411 ± 1.1319 | 0.3512 ± 0.6602 | 0.5535 ± 1.0953 |
| *Faecalibacteriu.* | species | 0.03 | 2.7024 ± 4.4598 | 3.9054 ± 3.8515 | 1.3642 ± 1.0295 | 1.3676 ± 1.1385 |
| *Ruminococcus gnavus* | species | 0.03 | 0.0160 ± 0.0231 | 0.0460 ± 0.0953 | 0.2743 ± 0.4642 | 0.7061 ± 1.7749 |
| *Lactobacillus* | species | 0.03 | 0.0803 ± 0.1010 | 0.1699 ± 0.2358 | 0.0814 ± 0.0883 | 0.0747 ± 0.1258 |
| *Clostridiu .scindens* | species | 0.03 | 0.0006 ± 0.0006 | 0.0005 ± 0.0006 | 0.0015 ± 0.0010 | 0.0004 ± 0.0005 |
| *Moryella* | species | 0.03 | 0.0335 ± 0.0223 | 0.0508 ± 0.0569 | 0.0904 ± 0.0921 | 0.0376 ± 0.0218 |
| *Papillibacter* | species | 0.04 | 0.0164 ± 0.0170 | 0.0299 ± 0.0290 | 0.0147 ± 0.0133 | 0.0055 ± 0.0057 |
| *Clostridium orbiscindens* | species | 0.04 | 0.2083 ± 0.2162 | 0.3174 ± 0.3328 | 0.6710 ± 0.5464 | 0.2628 ± 0.1674 |
| *Finegoldia magna* | species | 0.04 | 0.0004 ± 0.0013 | 0.0003 ± 0.0005 | 0.0070 ± 0.0126 | 0.0019 ± 0.0039 |
| *Anaerofilum* | species | 0.05 | 0.0033 ± 0.0034 | 0.0078 ± 0.0100 | 0.0023 ± 0.0034 | 0.0021 ± 0.0038 |
| *Blautia* | species | 0.05 | 2.5800 ± 1.8870 | 3.5056 ± 1.8120 | 10.8724 ± 9.8969 | 6.1710 ± 6.7715 |
| *Sporobacter* | species | 0.05 | 0.0173 ± 0.0083 | 0.0311 ± 0.0263 | 0.0071 ± 0.0151 | 0.0024 ± 0.0033 |
| *Dehalobacterium* | species | 0.05 | 0.0079 ± 0.0103 | 0.0238 ± 0.0287 | 0.0041 ± 0.0085 | 0.0009 ± 0.0009 |

**Supplementary table 3: The list of bacterial taxa with significant differences in the mean change from baseline in Pre-DM subjects**

| **Taxonomic assignment** | | ***p*-value** | **Abundance** | | | |
| --- | --- | --- | --- | --- | --- | --- |
| **Name** | **level** |  | **Placebo W0** | **Placebo W8** | **XOS W0** | **XOS W8** |
| *Fusobacteria* | class | 0.02 | 0.0062 ± 0.0065 | 0.0010 ± 0.0012 | 0.0005 ± 0.0010 | 0.0009 ± 0.0020 |
| *Gammaproteobacteria* | class | 0.04 | 0.2793 ± 0.3902 | 0.0257 ± 0.0194 | 0.0192 ± 0.0313 | 0.0077 ± 0.0098 |
| *Coriobacteria* | class | 0.05 | 0.1667 ± 0.1270 | 0.2224 ± 0.1926 | 0.2054 ± 0.1635 | 0.1128 ± 0.0853 |
| *Veillonellaceae* | family | 0.04 | 13.1701 ± 9.2057 | 9.8555 ± 14.0910 | 3.6640 ± 5.6319 | 9.7636 ± 11.2680 |
| *Oscillospira* | genus | 0.01 | 5.3576 ± 5.2097 | 2.9783 ± 3.7637 | 1.5301 ± 1.2988 | 3.5768 ± 2.8753 |
| *Dialister* | genus | 0.01 | 8.4033 ± 7.1893 | 2.3356 ± 2.7122 | 2.7262 ± 4.7304 | 6.9532 ± 10.5424 |
| *Enterorhabdus* | genus | 0.01 | 0.0665 ± 0.1048 | 0.1162 ± 0.1548 | 0.0808 ± 0.0699 | 0.0293 ± 0.0351 |
| *Anaerotruncus* | genus | 0.02 | 1.0095 ± 0.5178 | 0.2790 ± 0.1749 | 0.2872 ± 0.2608 | 0.2651 ± 0.2809 |
| *Finegoldia* | genus | 0.03 | 0.0019 ± 0.0022 | 0.0002 ± 0.0005 | 0.0003 ± 0.0004 | 0.0008 ± 0.0014 |
| *Allobaculum* | genus | 0.04 | 0.0293 ± 0.0077 | 0.0237 ± 0.0035 | 0.0241 ± 0.0054 | 0.0260 ± 0.0049 |
| *Blautia* | genus | 0.05 | 9.0101 ± 5.8208 | 2.8773 ± 1.7101 | 3.3361 ± 1.7049 | 2.7252 ± 2.1776 |
| *Oscillospira* | species | 0.01 | 5.3576 ± 5.2097 | 2.9783 ± 3.7637 | 1.5301 ± 1.2988 | 3.5768 ± 2.8753 |
| *Dialister human gut* | species | 0.01 | 8.3487 ± 7.1574 | 2.3074 ± 2.6980 | 2.6967 ± 4.6853 | 6.8946 ± 10.4514 |
| *Dialister invisus* | species | 0.01 | 0.0427 ± 0.0230 | 0.0244 ± 0.0127 | 0.0280 ± 0.0440 | 0.0539 ± 0.0926 |
| *Clostridium scindens* | species | 0.01 | 0.0011 ± 0.0001 | 0.0005 ± 0.0006 | 0.0001 ± 0.0003 | 0.0008 ± 0.0001 |
| *Fusobacterium nucleatum* | species | 0.02 | 0.0059 ± 0.0068 | 0.0008 ± 0.0013 | 0.0001 ± 0.0003 | 0.0009 ± 0.0020 |
| *Eubacterium cylindroides* | species | 0.02 | 0.0000 | 0.0013 ± 0.0012 | 0.0020 ± 0.0035 | 0.0003 ± 0.0005 |
| *Anaerotruncus* | species | 0.02 | 1.0089 ± 0.5177 | 0.2786 ± 0.1744 | 0.2871 ± 0.2609 | 0.2650 ± 0.2807 |
| *Enterorhabdus* | species | 0.02 | 0.0665 ± 0.1048 | 0.1162 ± 0.1548 | 0.0807 ± 0.0701 | 0.0293 ± 0.0351 |
| *Dialister* | species | 0.03 | 0.0045 ± 0.0048 | 0.0007 ± 0.0008 | 0.0011 ± 0.0019 | 0.0033 ± 0.0050 |
| *Finegoldia magna* | species | 0.03 | 0.0019 ± 0.0022 | 0.0002 ± 0.0005 | 0.0003 ± 0.0004 | 0.0008 ± 0.0014 |
| *Dialister micraerophilus* | species | 0.04 | 0.0074 ± 0.0069 | 0.0031 ± 0.0037 | 0.0004 ± 0.0005 | 0.0015 ± 0.0017 |
| *Allobaculum* | species | 0.04 | 0.0293 ± 0.0077 | 0.0237 ± 0.0035 | 0.0241 ± 0.0054 | 0.0260 ± 0.0049 |
| *Ruminococcus bromii* | species | 0.05 | 1.3742 ± 2.8231 | 2.5782 ± 6.0290 | 1.7665 ± 2.6915 | 0.3555 ± 0.6846 |
| *Blautia* | species | 0.05 | 8.8470 ± 5.8415 | 2.7735 ± 1.5436 | 3.2523 ± 1.7153 | 2.7000 ± 2.1741 |
| *Desulfovibrio desulfuricans* | species | 0.05 | 0.0033 ± 0.0045 | 0.0047 ± 0.0042 | 0.0030 ± 0.0046 | 0.0025 ± 0.0042 |
| *Desulfovibrio piger* | species | 0.05 | 0.1668 ± 0.2553 | 0.3940 ± 0.6165 | 0.0677 ± 0.1422 | 0.0204 ± 0.0255 |
| *Blautia hydrogenotrophica* | species | 0.05 | 0.0007 ± 0.0008 | 0.0000 ± 0.0000 | 0.0004 ± 0.0010 | 0.0004 ± 0.0007 |

**Supplementary Table 4: Mean change of *Bifidobacterium* and related species from baseline in healthy subjects**

|  | ***Bifidobacterium (genus)*** | | | ***Bifidobacterium adolescentis (species)*** | | | ***Bifidobacterium longum (species)*** | | | ***Bifidobacterium bifidum (species)*** | | |
| --- | --- | --- | --- | --- | --- | --- | --- | --- | --- | --- | --- | --- |
| **Treatment** | **Mean** | **SD** | ***P*** | **Mean** | **SD** | ***P*** | **Mean** | **SD** | ***P*** | **Mean** | **SD** | ***P*** |
| placebo | -1.69 | 5.63 | 0.11 | -0.37 | 1.47 | 0.07 | -1.32 | 4.16 | 0.17 | 0.00 | 0.01 | 0.21 |
| XOS | 1.65 | 6.94 |  | -0.23 | 0.34 |  | 1.86 | 6.69 |  | 0.02 | 0.15 |  |
